# Lectures on the Principles and Practice of Surgery

**Published:** 1852-10

**Authors:** 


### BIBLIOGRAPHICAL NOTICES.

*Lectures on the Principles and Practice of Surgery.* By Bransby B. Cooper, F. R. S., Senior Surgeon to Guy's Hospital, &c. Philadelphia: Blanchard & Lea, 1852. 8vo. pp. 771, including Index.

